# Mapping combinatorial expression of non-clustered protocadherins in the developing brain identifies novel PCDH19-mediated cell adhesion properties

**DOI:** 10.1098/rsob.230383

**Published:** 2024-04-17

**Authors:** Stefka Mincheva-Tasheva, Chandran Pfitzner, Raman Kumar, Idha Kurtsdotter, Michaela Scherer, Tarin Ritchie, Jonas Muhr, Jozef Gecz, Paul Q. Thomas

**Affiliations:** ^1^ School of Biomedicine and Robinson Research Institute, University of Adelaide, Adelaide, South Australia 5005, Australia; ^2^ School of Medicine and Robinson Research Institute, University of Adelaide, Adelaide, South Australia 5005, Australia; ^3^ Genome Editing Program, South Australian Health and Medical Research Institute, Adelaide, South Australia 5000, Australia; ^4^ Department of Cell and Molecular Biology, Karolinska Institute, Stockholm, Sweden; ^5^ South Australian Health and Medical Research Institute, Adelaide, 5000 , Australia

**Keywords:** Protocadherin 19, PCDH19-clustering epilepsy, non-clustered protocadherins, cell adhesion, brain development

## Abstract

Non-clustered protocadherins (ncPcdhs) are adhesive molecules with spatio-temporally regulated overlapping expression in the developing nervous system. Although their unique role in neurogenesis has been widely studied, their combinatorial role in brain physiology and pathology is poorly understood. Using probabilistic cell typing by *in situ* sequencing, we demonstrate combinatorial inter- and intra-familial expression of ncPcdhs in the developing mouse cortex and hippocampus, at single-cell resolution. We discovered the combinatorial expression of Protocadherin-19 (*Pcdh19*), a protein involved in PCDH19-clustering epilepsy, with *Pcdh1*, *Pcdh9* or Cadherin 13 (*Cdh13*) in excitatory neurons. Using aggregation assays, we demonstrate a code-specific adhesion function of PCDH19; mosaic PCDH19 absence in PCDH19+9 and PCDH19 + CDH13, but not in PCDH19+1 codes, alters cell–cell interaction. Interestingly, we found that PCDH19 as a dominant protein in two heterophilic adhesion codes could promote *trans*-interaction between them. In addition, we discovered increased CDH13-mediated cell adhesion in the presence of PCDH19, suggesting a potential role of PCDH19 as an adhesion mediator of CDH13. Finally, we demonstrated novel *cis*-interactions between PCDH19 and PCDH1, PCDH9 and CDH13. These observations suggest that there is a unique combinatorial code with a cell- and region-specific characteristic where a single molecule defines the heterophilic cell–cell adhesion properties of each code.

## 1. Introduction

Brain development and neuronal network formation require strict coordination of neuronal migration and interaction. These processes are largely controlled by adhesion molecules, including the protocadherin (Pcdh) family [1–3]. Pcdhs are calcium-dependent cell adhesion molecules that are predominantly expressed during nervous system development and early post-natal stages [3]. Although PCDH functions in axon outgrowth, dendritogenesis, synaptogenesis and neuronal migration have been widely studied [4–8] and the effect of mutations in individual Pcdh genes linked to neurological phenotypes [1,4,9–14], it remains unclear how the absence of a single molecule, albeit in a cellular mosaic state, can alter cell–cell communication and brain function. Given the spatio-temporal overlapping expression of non-clustered Protocadherins (ncPCDH) family members *in viv*o [15], the combinatorial expression of these adhesion molecules, at a single-cell level, could play a critical role in the formation and maintenance of brain structural integrity [16]. On the other hand, missing a key molecule of a specific combinatory code could disrupt vital neuronal processes leading to a pathological phenotype [10,17].

Pcdhs are divided into clustered (cPcdhs), which are located in three genomic clusters, and ncPcdhs, which are scattered throughout the genome [14,18]. The 11 ncPcdhs are subdivided into three subgroups: δ1 (*Pcdh1, Pcdh7, Pcdh9* and *Pcdh11*), δ2 (*Pcdh8, Pcdh10, Pcdh17, Pcdh18* and *Pcdh19*) and δ0 (*Pcdh12* and *Pcdh20*), based on their homology and the number of extracellular cadherin (EC) repeats (EC1–7 in δ0 and δ1, and EC1–6 in δ2) [2,14,19,20]. ncPCDHs can promote cell–cell adhesion via *trans*-interaction of their conservative EC domains; two cells can generate an anti-parallel dimer via interaction between ECs in one cell and ECs in the other [21,22]. The *trans*-binding mechanism for δ1- and δ2-PCDHs tends to be homophilic [23,24] with weaker heterophilic interactions within subfamilies [20]. In addition, an intracellular *cis*-interaction between different ncPCDH family members has been demonstrated exclusively for PCDH19 [10].

Genomic variants in the X-linked *PCDH19* gene cause PCDH19-clustering epilepsy (PCDH19-CE), one of the most common forms of inherited epilepsy, which is characterized by variable seizures and incompletely penetrant intellectual disability [25–27]. PCDH19-CE has a unique mode of inheritance whereby heterozygous females and somatic mosaic males are affected while hemizygous males are clinically unremarkable [28]. The proposed mechanism underpinning this synular interference where the coexistence of two cell populations, expressing either WT or variant *PCDH19,* results in abnormal cellular interactions and neuronal communication in affected individuals [9,29]. Greater than 80% of PCDH19 disease variants are located in the region encoding for the EC domains, which are essential for homophilic interaction and cell–cell adhesion [30,31].

The aim of this study was to identify which ncPcdh family members are co-expressed with *Pcdh19* at single-cell resolution in the developing hippocampus and cortex and to functionally test their role in combinatorial cell adhesion. Using probabilistic cell typing by *in situ* RNA sequencing (psicRNAseq) [32], we found that *Pcdh19* was predominately co-expressed with *Pcdh1*, *Pcdh9* or *Cdh13* in *Gria2*+ glutamatergic excitatory neurons in developing mouse brain. Using cell aggregation assays, we demonstrated a critical role for PCDH19 in PCDH19+9 and PCDH19 + CDH13 combinatorial codes, suggesting that the mosaic absence of PCDH19 in the central nervous system (CNS) of PCDH19-CE-affected individuals could alter interactions of cells expressing these codes. Collectively, this study defines the spatial distribution of the Pcdh19–ncPcdhs combinatorial expression in the developing cortex and hippocampus and provides new insight into PCDH19-mediated cell–cell adhesion.

## 2. Results

### 2.1. Identifying *Pcdh19* combinatorial expression at single-cell level in the developing mouse brain

**Figure 1.**
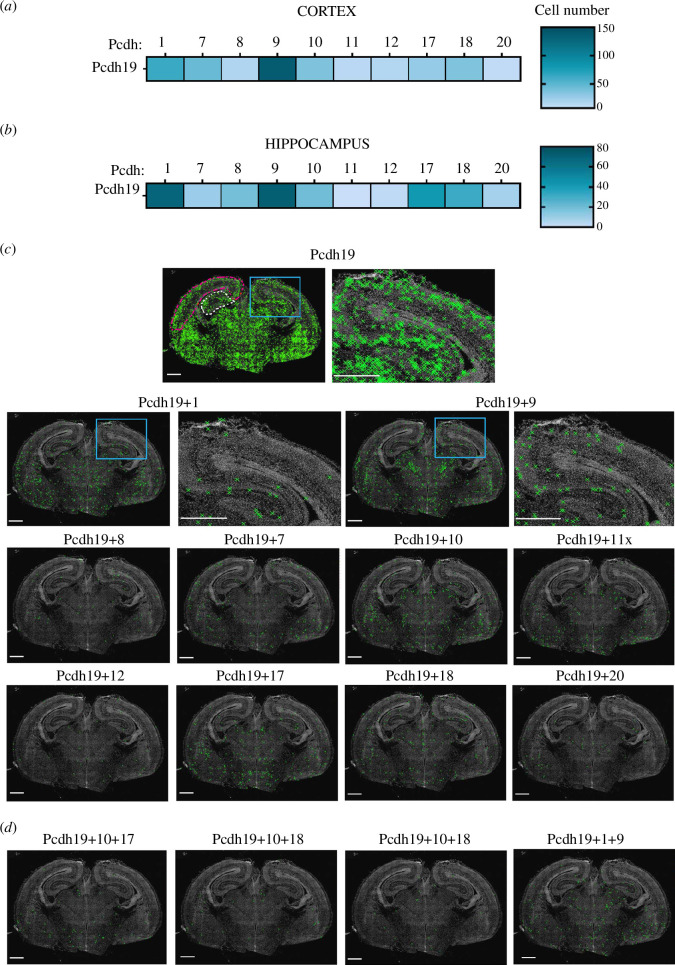
Expression profile of *Pcdh19* + ncPcdhs in mouse cortex and hippocampus. Heat map of the mean number of cells co-expressing *Pcdh19* and ncPcdhs in the cortex (*a*) and hippocampus (*b*). Each row represents the number of cells co-expressing *Pcdh19* and an individual member of ncPcdhs (*Pcdh1, 7, 8, 9, 10, 11, 12, 17, 18* and *20*). (*c*) Representative phase contrast images of *Pcdh19*-only and co-expressing *Pcdh1* or *7, 8, 9, 10, 11, 12, 17, 18* or *20* cells in a mouse brain at 18.5 dpc. The cortical areas highlighted in magenta and the hippocampal areas highlighted in white considered for the analysis in (*a*) and (*b*). (*d*) Representative images of *Pcdh19* positive cells in the brain that co-express *Pcdh10+17*, *Pcdh10+18*, *Pcdh17+18* and *Pcdh1+9*. The green ‘X’ in (*c*) and (*d*) represents the position of Pcdh-expressing cells in the brain based on the DAPI signal. Scale bar 500 µm.

ncPcdhs are widely expressed in CNS during development and in adulthood [18,33] (electronic supplementary material, figure S1a). Although it has been recently proposed that a single cell can express more than one Pcdh [10,17], cell-specific co-expression of these molecules in the cortex and hippocampus *in vivo* is yet to be determined. Using the *in situ* seq method, we investigated co-expression of the 11 ncPcdhs in the developing mouse brain (18.5 dpc) at single-cell resolution (figure 1*a–c*). Consistent with previous reports, we found that ncPcdhs are highly expressed in mouse brain [4,10,34] (electronic supplementary material, figure S1a). We observed that a high number of cells in the cortex and hippocampus express *Pcdh1* or *Pcdh9*; a medium–high number of cells express *Pcdh7*, *8*, *10*, *17*, *18* or *19*, whereas *Pcdh11*, *12* or *20* were detected only in a limited number of cells (electronic supplementary material, figure S1a).

Next, we investigated the co-expression of *Pcdh19* and the other 10 ncPcdhs. In the cortex and hippocampus, *Pcdh19* was co-expressed with most other ncPcdh family members, although the number of cells expressing a specific combination was highly variable (figure 1*a*–*c* and electronic supplementary material, figure S2a,b). Interestingly, in these brain regions, the highest number of cells co-expressed *Pcdh19* and *Pcdh9*, and *Pcdh19* and *Pcdh1* (figure 1 and electronic supplementary material, figure S2), whereas only a relatively moderate number of cells expressed *Pcdh19* together with its previously identified partners, *Pcdh17* and *Pcdh18* [10].

To further characterize the *Pcdh19* co-expression code in the cortex and hippocampus, we next evaluated the number of other ncPcdhs expressed in *Pcdh19* positive cells. A very low number of cells expressed *Pcdh19* and two additional ncPcdhs, and no cells were found to express *Pcdh19* and three or more ncPcdhs (figure 1*d*). These observations suggest that in the developing cortex and hippocampus, *Pcdh19* combinatorial expression is mostly represented by only one additional ncPcdh.

**Figure 2.**
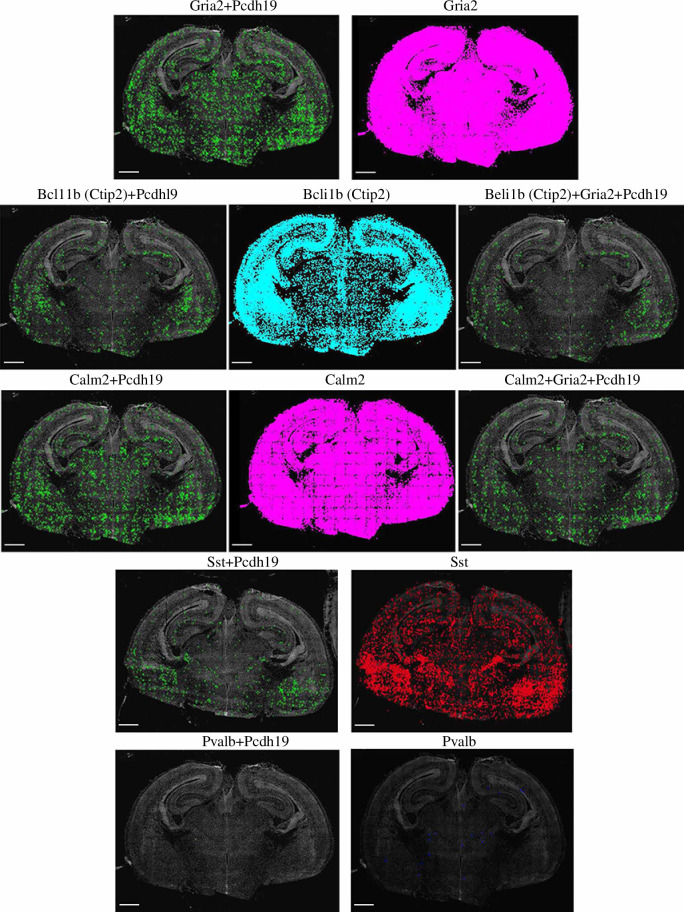
Pcdh19 expression in brain excitatory and interneurons. Representative *in situ* RNA seq images of coronal mouse brain sections at 18.5 dpc with *Gria2* (magenta), *Ctip2* (Bcl11b) (cyan), *Calm2* (magenta), label excitatory neurons; *Sst* (red) and *Pvalb* (blue) to label interneurons, with (left panels) or without *Pcdh19* (right panels) (*Pcdh19* + cell markers), the green ‘X’ represents the position of these cells based on the DAPI signal). Scale bar 500 µm.

**Figure 3.**
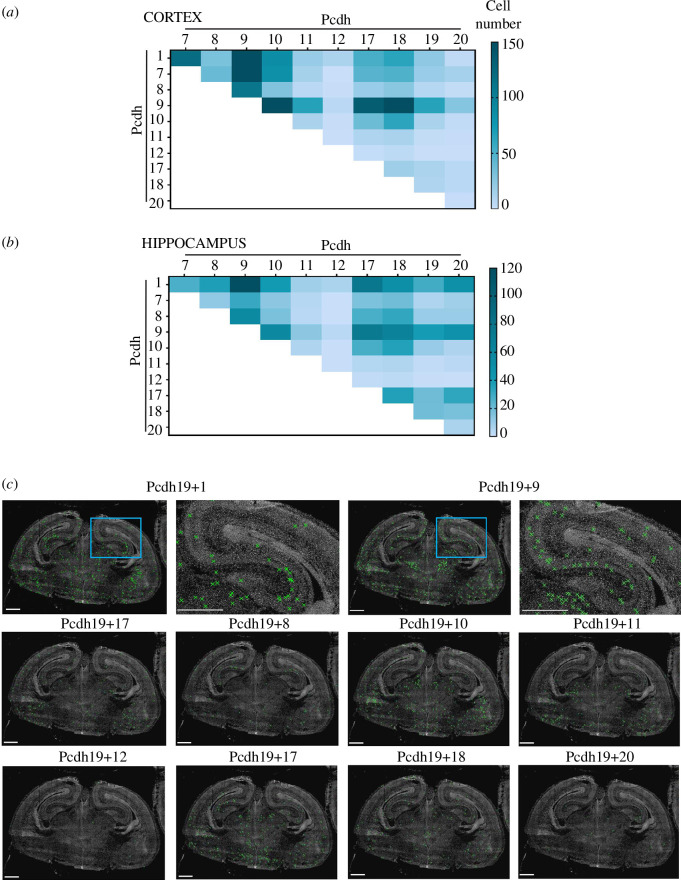
Co-expression of ncPcdhs in glutamatergic cortical and hippocampal excitatory neurons during brain development. Heat map of the mean number of cells co-expressing ncPcdh in glutamatergic (*Gria2* positive) cortical (*a*) and hippocampal (*b*) neurons. Each row represents the number of cells co-expressing any two of *Pcdh1, 7, 8, 9, 10*, *11, 12, 17, 18, 19* and *20*. (*c*) Representative phase contrast *in situ* RNA seq images of coronal mouse brain sections at 18.5 dpc labelling *Gria2* positive cells that co-express *Pcdh19*+ncPcdhs (*Pcdh1 7, 8, 9, 10, 11, 12, 17*, *18, 19 or 20*). The green ‘X’ represents the position of these cells based on the DAPI signal. Scale bar 500 µm.

To determine the neural cell types that express the *Pcdh19* combinatorial code, we used expression markers to identify excitatory neurons (B-cell lymphoma/leukemia11B (*Bcl11b*/*Ctip2*), Calmodulin 2 (*Calm2*) and Glutamate Ionotropic Receptor AMPA Type Subunit 2 (*Gria2*)), interneurons (Somatostatin (*Sst*) and Parvalbumin (*Pvalb*)) and astrocytes (Aquaporin 4 (*Aqp4*) and Glial fibrillary acidic protein (*Gfap*)) (figure 2 and electronic supplementary material, figure S1b). *Pcdh19* is predominantly expressed in glutamatergic *Gria2* neurons. Commonly, excitatory neuronal subtypes express more than one cellular marker. Here, we have found that *Pcdh19*-expressing cells are positive for at least two excitatory neuronal markers (figure 2). Because the highest number of neurons expressing *Pcdh19* are *Gria2* positive, we then investigated the combinatorial expression of *Pcdh19* and other ncPcdhs in this glutamatergic neuronal subtype (figure 3*a*,*b*). In cortical excitatory neurons, *Pcdh19* was predominately co-expressed with *Pcdh9*. In addition, *Pcdh1*, *7*, *10*, *17* or *18* were also co-expressed with *Pcdh19* in a moderate–low number of cells, whereas *Pcdh19+8*, *11*, *12* or *20* were only detected in a few isolated excitatory neurons (figure 3*a*,*c* and electronic supplementary material, figure S3a). In hippocampal glutamatergic neurons, *Pcdh19* was mostly co-expressed with *Pcdh9* and *Pcdh1*. A medium–low number of cells were found to co-express *Pcdh19* with *Pcdh7*, *8*, *10*, *17* or *18* (figure 3*b*,*c* and electronic supplementary material, figure S3b). We also investigated combinatorial *Pcdh19* expression in interneurons and astrocytes (figure 2 and electronic supplementary material, figure S1b). Co-expression of *Pcdh19* with other ncPcdh(s) was identified in a few *Sst*-positive interneurons, but not in *Pvalb* interneurons or astrocytes (*Gfap+* or *Aqp4+*) (electronic supplementary material, figure S1a,b). Collectively, these observations reveal a region-specific and cell type-specific expression of *Pcdh19*+ncPcdhs combinatorial code.

### 2.2. Combinatorial expression of *Pcdh19* and *Cdh13* in cortex and hippocampus

**Figure 4.**
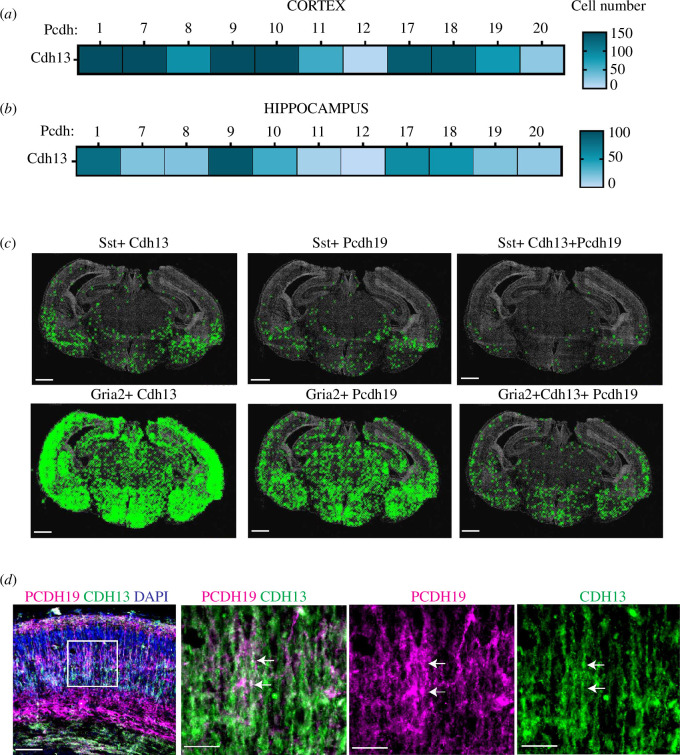
*Pcdh19* expression in *Cdh13* positive neurons. Heat map of mean number of cells co-expressing *Cdh13* and ncPcdhs in cortex (*a*) and hippocampus (*b*), at E18.5. Each row represents the number of cells co-expressing *Cdh13* and *Pcdh1, 7, 8, 9, 10, 11, 12, 17, 18, 19* or *20*. (*c*) Representative phase contrast *in situ* RNA seq images of coronal mouse brain sections at 18.5 dpc labelling *Sst*-positive (top) or *Gria2* positive (bottom) neurons expressing *Cdh13* (left) or *Pcdh19* (middle) and *Pcdh19 + Cdh13* (right). The green ‘X’ represents the position of these cells based on the DAPI signal. Scale bar 500 µm. (*d*) Representative images of coronal brain sections of *Pcdh19*-HA/Flag Tagged mouse at 18.5 dpc immunostained with anti-CDH13 (green), anti-HA (PCDH19) (magenta) and DAPI (blue). The arrows indicate the co-localization (white) of CDH13 and HA. Scale bars: PCDH19+CDH13+DAPI is 100 µm; PDH19+CDH13, CDH13 and PCDH19 are 50 µm.

CDH13 is expressed at very high levels in the developing cortex and hippocampus during embryogenesis and has been shown to play a role in neurogenesis and synaptogenesis [35]. *In situ* RNA seq analysis showed that in the cortex a high number of cells co-express *Cdh13* and ncPcdhs (figure 4 and electronic supplementary material, figure S4). Interestingly, although *Pcdh19* is expressed in a moderate number of *Cdh13*-positive cells in the cortex (figure 4*a*), in the hippocampus, only a few isolated cells express *Pcdh19 + Cdh13* (figure 4*b*). Analysis of the cell type expressing *Cdh13* and *Pcdh19* in the cortex and hippocampus showed that *Pcdh19 + Cdh13* are mostly co-expressed in *Gria2*-positive excitatory neurons (figure 4*c*). To further compare *Pcdh19* and *Cdh13* expression, we performed co-immunostaining of CDH13 and PCDH19 using our previously established *Pcdh19-*HA/Flag mouse model [10]. Extensive co-localization of PCDH19 (HA) and CDH13 was detected in the developing cortex (figure 4*d*), consistent with our *in situ* RNA seq data.

### 2.3. Unique adhesion function of PCDH19 in PCDH19 + CDH13 and PCDH19+9 combinatorial codes

We and others have shown that cell populations expressing multiple ncPCDH can only completely intermix if they express the same combination of PCDH molecules and that cells expressing a ‘partially overlapping code’ cannot [10,17]. Prompted by our *in vivo* co-expression data, we investigated the combinatorial adhesion properties of PCDH19 with PCDH1 or PCDH9 using K562 cells (which do not express endogenous cadherins) [10,17]. Aggregation assays were performed by overexpressing PCDH19 with PCDH1 or PCDH9 with green fluorescent protein (GFP) and mCherry (MCH) fluorescent reporters to label each cell population (electronic supplementary material, figure S5).

**Figure 5.**
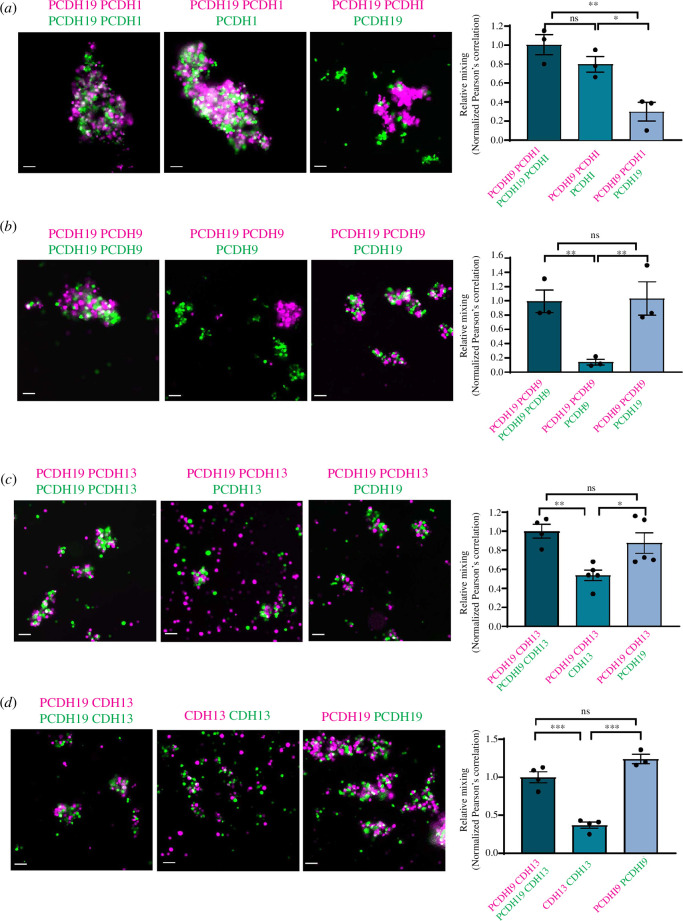
Heterophilic *trans*-interactions between PCDH19 + ncPCDHs. Representative images of mismatch aggregation assay performed by mixing cells expressing (*a*) PCDH19+1 with cells expressing PCDH19 or PCDH1, (*b*) PCDH19+9 with cells expressing PCDH19 or PCDH9 only, (*c*) PCDH19 + CDH13 with cells expressing PCDH19 or CDH13 only or (*d*) comparing cells expressing PCDH19 + CDH13 with PCDH19 or CDH13 only. Quantification of the degree of mixing using Pearson’s correlation coefficient normalized to PCDH19+1 and PCDH19+1 (*a*) or PCDH19+9 and PCDH19+9 (*b*), PCDH19 + CDH13 (*c* and *d*), respectively (^∗^*p* < 0.05; ^∗∗^*p* < 0.01; ^∗∗∗^*p* < 0.001; ns, not significant; one-way ANOVA with Tukey’s multiple comparison test against). Scale bar 100 µm. Error bars represent the mean ± standard error (s.e.m.).

Cell aggregates were evaluated as follows: aggregates mainly formed by one population indicated cell sorting, whereas the presence of heterogeneous populations indicated cell mixing (intermixing) (electronic supplementary material, figure S5) [10]. Firstly, to confirm aggregation activity, we compared cell populations expressing a single identical PCDH molecule: PCDH19 (MCH) + PCDH19 (GFP), PCDH1 (MCH) + PCDH1 (GFP) or PCDH9 (MCH) + PCDH9 (GFP) (figure 5*d* and electronic supplementary material, figure S5). As expected, extensive cell mixing was observed in all cases. In contrast, when we mixed cells expressing PCDH19 (MCH) + PCDH1 (GFP), PCDH19 (MCH) + PCDH9 (GFP), cell sorting was observed (electronic supplementary material, figure S5).

To investigate the PCDH combinatorial activity, we performed mismatch co-aggregation assays in which populations of K562 cells expressing PCDH19+1 or PCDH19+9 were mixed with cells expressing individual proteins of each combination (figure 5*a*,*b*). To quantitate the degree of cell mixing, we performed Pearson’s correlation coefficient analysis [10]. The maximum level of intermixing (100%) was established using cells expressing the same combination of adhesion molecules: PCDH19+1(GFP) and PCDH19+1(MCH) or PCDH19+9(GFP) and PCDH19+9(MCH). Firstly, we assessed the PCDH19 adhesion function in PCDH19+1 combinatorial code (figure 5*a*). An approximately 70% decrease in relative mixing was observed between cells expressing the complete code (PCDH19+1) and those expressing PCDH19 only. However, no significant difference in the level of relative mixing was detected between cells expressing PCDH19+1 and PCDH1 only (figure 5*a*).

Next, we investigated the role of PCDH19 in PCDH19+9 combinatorial code. A significant (approx. 85%) reduction in relative mixing was observed between populations expressing the complete code (PCDH19+9) and those expressing PCDH9 only (figure 5*b*). In contrast, relative mixing between cells expressing PCDH19+9 and PCDH19 was not significantly affected (figure 5*b*). Collectively, these observations indicate a 'dominant' PCDH19 adhesion role in PCDH19+9, but not in PCDH19+1 combinatorial codes.

Next, we evaluated the combinatorial CDH13+PCDH19 activity by performing mismatch co-aggregations assays as described above. Two populations of fluorescently labelled K562 cells expressing identical (PCDH19+CDH13 and CDH13+PCDH19) or different (PCDH19+CDH13 and PCDH19) or (PCDH19+ CDH13 and CDH13) combinations were mixed (figure 5*c*). Significant sorting between PCDH19+CDH13 and CDH13 compared with control (PCDH19 + CDH13 and PCDH19 + CDH13) was observed. In contrast, cell populations expressing PCDH19+CDH13 and PCDH19 did not show a significant decrease in cell mixing compared with control (figure 5*c*). Taken together these observations indicate that mosaic absence of PCDH19 in PCDH19+9 and PCDH19+CDH13, but not in PCDH19+1 codes significantly alter cell–cell interaction.

To investigate the adhesion properties of PCDH19 in combination with CDH13, we performed aggregation experiments using K562 cells (figure 5*d*). Firstly, we compared cell populations expressing a single PCDH19 and CDH13 molecule labelled with different fluorescent markers (MCH/GFP) as described above. Extensive sorting was observed when cells expressing either PCDH19 or CDH13 were mixed (electronic supplementary material, figure S5). In contrast, combining cell populations expressing PCDH19+CDH13 showed extensive mixing confirming homotypic cell–cell adhesion activity of both adhesion proteins (figure 5*d*). Interestingly, CDH13 alone showed low cell–cell adhesion; however, its co-expression with PCDH19 significantly increased cell aggregation (figure 5*d*).

**Figure 6.**
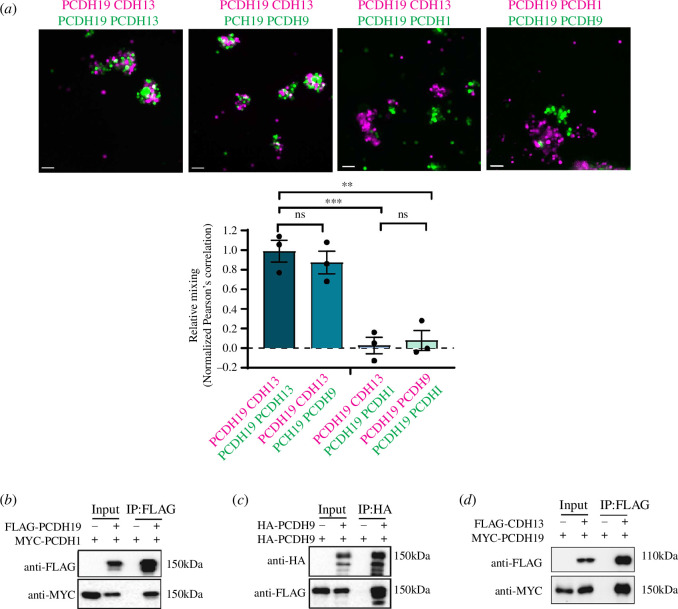
Heterophilic *trans-* and *cis*-interactions between PCDH19 and ncPCDHs reveals novel PCDH19 adhesion properties. (*a*) Representative images of aggregation assay performed with K562 cells by mixing cells expressing: PCDH19 + CDH13 & PCDH19 + CDH13 (GFP & MCH), CDH19 + CDH13 & CDH19 + PCDH9 (MCH & GFP), PCDH19 + CDH13 & PCDH19 + PCDH1 (MCH & GFP) or CDH19 + PCDH1 & PCDH19 + PCDH9 (MCH & GFP). Graph represents quantification of the degree of mixing using Pearson’s correlation coefficient normalized to PCDH19 + CDH13 & PCDH19 + CDH13 (^∗∗^*p* < 0.01, ^∗∗∗^*p* < 0.001; ns, not significant; one-way ANOVA with Tukey’s multiple comparison test). Scale bar 100 µm. Error bars represent the mean ± standard error (s.e.m.). Co-immunoprecipitation of HEK293T cell lysates expressing PCDH19-FLAG and PCDH1-MYC (*b*), PCDH9-HA and PCDH19-FLAG (*c*), and CDH13-FLAG and PCDH10-MYC (*d*), combinations indicated by the ‘+” and ‘−” symbols. Lysates were pulled down with FLAG (*b* and *d*) or HA (*c*) antibody and subsequent blotting was performed with anti-MYC and anti-FLAG (*b*), anti-HA and anti-FLAG (*c*) or anti-FLAG and anti-MYC (*d*).

Next, we investigated whether PCDH19 as a key adhesion protein in two different combinatorial codes could promote a heterophilic interaction between them. We mixed two populations of fluorescently labelled K562 cells expressing PCDH19+CDH13 with PCDH19+PCDH9 (figure 6*a*). No significant difference in cell mixing was observed compared with control (PCDH19+CDH13 and PCDH19+CDH13). We also performed a reciprocal experiment where we mixed cells expressing combinatorial codes with different 'dominant' adhesion proteins: PCDH19+CDH13 or PCDH19+PCDH9 (PCDH19 is the key adhesion protein) with PCDH19+PCDH1 (PCDH1 is the key adhesion protein) (figure 6*a*). Near complete segregation of PCDH19+9 or PCDH19+CDH13 and PCDH19+1 cells versus control cells was observed. Altogether these observations suggest that the heterophilic adhesion between two different cell populations could occur only if the same dominant ncPCDH (PCDH19) is present.

Cadherins have previously been shown to undergo *cis-* and *trans*-interactions at the interface of adjacent cells [36]. To evaluate PCDH19 *cis*-interaction with PCDH9, PCDH1 and CDH13, we performed immunoprecipitation analysis of differentially tagged proteins expressed in HEK293T cells (figure 6*b–d*). Our results revealed that PCDH19 could interact with both δ1-PCDHs and CDH13. Taken together these observations suggest that despite the similar *cis*-interaction between PCDH19 and PCDH1/PCDH9/CDH13, each individual combinatorial code presents *trans* sensitivity only to a specific ncPCDH.

## 3. Discussion

Overlapping expression and spatial distribution of ncPCDHs support their potential combinatorial contribution to brain architecture and physiology [16–18,37]. Although we [10] and others [17,18] were able to demonstrate the co-expression of ncPcdhs in the brain, the complexity of this expression code at single-cell resolution across specific brain regions remains poorly understood. Using *in situ* RNA sequencing *in vivo,* we have found that most cortical and hippocampal excitatory neurons express two ncPcdhs (δ1 + δ2 or δ1 + δ1). Consistent with our observations, a recent study performed by Bisogni *et al.* revealed that olfactory neurons also mostly express two ncPcdhs, although some of these cells could express up to seven [17], something that we have not observed in the cortex and hippocampus. In contrast to our study, Bisogni *et al*. used the NanoString nCounter platform to identify ncPcdh combinatorial expression in post-natal olfactory neurons. Thus, the greater complexity of Pcdh expression identified by Bisogni *et al*. may reflect a difference in technical sensitivity or developmental stage specificity of ncPcdh expression. Another potential explanation is that the number of ncPcdhs expressed per cell is region-specific, perhaps reflecting requirements for local circuit formation and/or maintenance [15].

Pcdhs have been implicated in multiple steps in excitatory neuronal circuit formation [1]. In the present study, we focus on *Pcdh19*, a causative gene of PCDH19-CE, hypothesizing that perturbation of Pcdh19-associated combinatorial adhesion codes could compromise CNS function in affected heterozygous females. We have identified prevalent expression of *Pcdh19+9* and *Pcdh19+1* in the hippocampus and cortex which correlates with the relatively high expression of these proteins in the brain. Considering that PCDH9 and 19 are found in excitatory neuron synapses and variants in their cognate genes have been linked to neurodevelopmental disorders [9,29,38], it is possible that they control similar neurocircuit processes. In contrast, besides the higher expression of *Pcdh1* in the mouse brain, mutations in *PCDH1* have been exclusively linked to respiratory disorders in humans [39] suggesting a potential auxiliary cell adhesion role in the CNS, perhaps through other PCDHs.

It has been previously reported that *Pcdh19* is expressed in a relatively high number of excitatory and interneurons, as well as in astrocytes at post-natal stages [40]. In the developing brain, although *Pcdh19* is expressed only in glutamatergic neurons and in some *Sst*-positive interneurons, but not in *Pvalb*-positive interneurons or astrocytes. Importantly, *Pcdh19+*ncPcdhs combinatorial expression was observed only in excitatory neurons, but not in *Sst* interneurons. To date, there is no evidence to support the existence of a combinatorial ncPcdh adhesion code in interneurons, perhaps reflecting a role for combinatorial expression of other cadherins, or even the complete lack of adhesion code in this cell type. Hence, the determination of combinatorial adhesion code in this cell type requires further investigation. On the other hand, the lack of *Pcdh19* expression in both astrocytes and *Pvalb* interneurons could be due to the lower prevalence of these cell subtypes during brain development or the complete absence of *Pcdh19* expression in these cells throughout embryogenesis.

We [10] and others [17] have shown that missing a single protein in a PCDH combinatorial code significantly reduces intermixing with cells expressing the complete code. Here, we demonstrate that this is not always the case; for example, cells expressing PCDH19+9 completely intermix with cells expressing PCDH19 only. Similar results were reported by Bisogni *et al*., where the authors demonstrated that populations expressing the complete code in some cases could still intermix with cells expressing only a dominant protein [17]. We have also shown that a key protein in one combinatorial code does not necessarily play a dominant role in a different combinatorial environment. However, considering the affinity differences and/or relative expression levels of ncPCDHs [17] mismatch aggregation assay may not comprehensively address these behaviours. In the case of PCDH19+1, missing PCDH1, but not PCDH19 has a significant impact on cell–cell adhesion between cells expressing the complete code. This finding may reflect a level of context-dependent redundancy in the combinatorial role of PCDH family members during CNS development in neuronal circuit assembly and maintenance. Interestingly, we have found that intermixing between cells expressing PCDH19 plus different adhesion molecules could occur only if PCDH19 is the dominant protein in both codes, as we have observed in the case of PCDH19+CDH13 and PCDH19+9. This finding could explain why missing a single adhesion protein causes such a diversity in the physiological outcome in a cell-specific manner. Considering that most of the neurons in the brain express several PCDHs and CDHs, the lack of one protein could be potentially compensated by other adhesion molecules. However, depending on whether a dominant protein of a combinatorial code is missing, will define whether the neuronal communication and brain circuit may be impacted.

*Cis*-interaction between PCDHs has been widely studied in cPCDHs; however, little is known about such interactions in ncPCDHs. It has been proposed for clustered and ncPCDHs that generate specific cell surface interaction codes using a ‘zippering’ mechanism where *cis* dimers of PCDHs from one cell interact in *trans* with complementary dimers from a second cell in antiparallel orientation [20,21]. Interestingly, besides the structural differences, it seems that ncPCDHs behave in a similar way. To date, only PCDH10 and PCDH17 have been shown to interact in *cis* with PCDH19 [10]. Although a recent biophysical study of EC domains of ncPcdhs has shown the lack of strong *cis*-interactions between members of the family [20], here using co-immunoprecipitation assays we were able to demonstrate an intracellular (*cis*) interaction between full-length ncPCDHs possessing a different number of EC domains: PCDH1 and PCDH19, and PCDH9 and PCDH19. Importantly, Harrison and collaborators focused on EC domains only, and in their original work, the authors do not exclude the possible contribution of other (transmembrane or cytoplasmic) domains in the establishment of these interactions [4,20].

In the last decade, it has been proposed that there is a close functional relationship between δ2-Pcdh members and classical cadherins [7,41]. Intriguingly, PCDH19 has been found to interact with N-cadherin (NCAD) regulating cell movement during morphogenesis in zebrafish [42]. In addition, a *cis*-interaction between PCDH19 and NCAD was proposed to be involved in hippocampal presynaptic function and cognitive behaviour [43]. Here, we have shown that *Pcdh19* and *Cdh13* are highly co-expressed in glutamatergic cortical neurons and that these proteins can interact in *cis*. In the nervous system, CDH13 together with PCDH19 is found in synaptic space controlling vital neuronal processes including axonal outgrowth and synaptic formation, consistent with their genetic contribution to a number of neurodevelopmental disorders [4,9,29,44,45].

Interactions between ncPcdhs and cadherins have been shown to modulate their adhesion abilities [42]. For instance, the lack of *Pcdh19* or *Ncad* negatively affects cell adhesion. Importantly, CDH13 protein lacks the cytoplasmic domain characteristic of other cadherins, suggesting that its adhesion abilities could be promoted by additional interactors. Here, we have found that *cis* (intracellular) interaction between CDH13 and PCDH19 can impact CDH13 adhesion, as loss of PCDH19 in the same cell affects CDH13 cell–cell aggregation abilities suggesting that ncPCDHs and cPCDHs interactions may play a role in the modification of cell–cell adhesion properties *in vivo*.

It has been shown that mosaic expression of PCDH19 disrupts neuronal communication in PCDH19-CE [9,43]. The critical role of PCDH19 in PCDH19+9 and PCDH19+CDH13 combinatorial codes suggests that missing PCDH19 in the mosaic population could disturb the adhesion properties between the cells expressing the complete code and the PCDH19 mutant cells expressing CDH13 or PCDH9 only. Considering the synaptic localization of PCDH9, CDH13 and PCDH19 [4,5,9,46–48], a mismatch in expression could alter the synaptic contact adhesion leading to network dysfunction and cognitive impairment, both of which are features of PCDH19-CE pathology.

## 4. Material and methods

### 4.1. Experimental animals

*In situ* sequencing was performed using CD1 mice (Jackson Laboratory) housed at Karolinska Institute, Sweden, and all the procedures and experiments were performed in accordance with Swedish animal welfare laws authorized by the Stockholm Animal Ethics Committee: DNR 3796-2020.

The animals used for co-immunostaining of PCDH19 and CDH13 were housed at the South Australian Health and Medical Research Institute, Australia, and the experiments were conducted under SAM20-015. Experiments were performed using a previously established *Pcdh19*-Tag mouse model [10].

#### 4.1.1. *In situ* sequencing and immunostaining of mouse samples

Brain samples (18.5 dpc) were cryosectioned coronally at 10 μm (mouse brain atlas position 250–276), mounted onto Super Frost plus microscope slides (Thermo Fisher Scientific) and stored at −80°C. The slides were then transferred on dry ice to CARTANA (Solna, Sweden; now part of 10× genomics) for tissue fixation and *in situ* sequencing. The reverse transcription, probe ligation, rolling cycle amplification with reagents and according to the procedures supplied in the Neurokit (1010-01, CARTANA, Sweden) were performed at CARTANA followed by fluorescence labelling, and sequencing by sequential images. One probe was designed for each ncPcdh gene: *Pcdh1, 7, 8, 9, 10, 11x, 12, 17, 18, 19* and *20*, and *Cdh13*, and each cellular marker: *Gira2, Calm4, Bcl11b* (Ctip2), *Stt, Pvalb, Aqp4* and *Gfap* (electronic supplementary material, figure S6). The data were collected over six rounds of five-colour microscopy images. The original images are taken using 40× objective. The result table of the spatial coordinates of each molecule of target genes together with the reference DAPI image per sample were provided by CARTANA. A customized script was used for data analysis from the original CARTANA script to approximate the locations of cells and assign individual CARTANA reads to specific cells.

#### 4.1.2. *In situ* sequencing imaging analysis for cell type mapping

Cellular markers for excitatory neurons (*Gria2, Calm2* and *Bcl11b*), inhibitory neurons (*Sst* and *Pvalb*) and astrocytes (*Gfap* and *Aqp4*) were used to define the cellular population expressing a single or combination of ncPCDHs. Each cellular marker was tested for expression of a combination of all 12 ncPcdhs simultaneously. The total number of cells expressing a combination of each of the two Pcdhs was analysed in the whole cortical and hippocampal areas/slice (left and right hemisphere) in each of the two sections per mouse (three mice in total). For generic co-expression of *Pcdh19* and single members of ncPcdhs in the brain, *Pcdh19* was used as a selection marker (figure 1). For expression of *Cdh13* and single members of ncPcdhs, *Cdh13* expressing cells were used as a selection marker. The data are presented as a heat map as an average of the three samples (two sections/mouse). The number of cells in the cortex expressing ncPcdhs and/or *Cdh13* were counted and represented as an average of six samples. Due to a very close proximity and very high number of *Gria2*+ neurons expressing *Pcdh1+9*, *Pcdh7+9, Pcdh9+10* or *Pcdh9+18*, and *Cdh13* co-expressing neurons and *Pcdh1, Pcdh9, Pcdh7* or *Pcdh10* per brain (data not shown), their values are indicated as greater than 150 in the graphs in electronic supplementary material, figures S3 and S4.

### 4.2. Immunofluorescence of brain slices

E18.5 mouse brains were fixed in 4% PFA at 4°C overnight as previously described [49]. Brains were cryoprotected in 30% sucrose and frozen in the Optimum Cutting Temperature embedding medium. Then 16 µm sections were prepared using a Leica CM1900 cryostat. Sections were blocked with 0.1% Triton X-100 and 10% horse serum in 1× PBS for 1 h at room temperature. Sections were then incubated overnight with rabbit anti-HA antibody 1:400 (Sigma) and anti-CDH13 1:300 (In Vitro Technologies) at 4°C. Slices were then washed three times with PBS and incubated with secondary donkey anti-rabbit Alexa 594 and anti-chicken 488 (Jackson ImmunoResearch) antibodies for 2 h at room temperature. Slides were mounted in Prolong™ Gold Antifade with DAPI (Invitrogen #P36931), and the images were acquired on a Nikon Eclipse Ti microscope and Nikon Digital Sight DS-Qi1 camera at 20×.

### 4.3. Generation of overexpression plasmids

To overexpress Pcdh1, Pcdh9 and Cdh13 in K562 and HEK293T cells, a full-length mouse, Pcdh19-Myc or Pcdh19-Flag, Pcdh1-Myc (NM_029357.3), Pcdh9-HA (NM_001081377.3) and Cdh13-Flag (NM_019707.5) were cloned into EF1A-CMV-GFP and EF1A-CMV-MCH plasmids (Victor Builder [10]) to generate into EF1A-PCDH19-CMV-GFP/MCH, EF1A-PCDH1-CMV-GFP/MCH, EF1A-PCDH9-CMV-GFP/MCH and EF1A-CDH13-CMV-GFP/MCH constructs.

### 4.4. K562 mixing assay and cell aggregation imaging

Totally, 2 × 10^6^ K562 cells were nucleofected as previously described in [10]. Briefly, the cells were transfected using 5 μg of each plasmid DNA (10 μg total for co-nucleofections) and incubated for 24 h. Cells were harvested and centrifuged at 170*g* for 7 min, then the pellets were resuspended in culture media to obtain a single-cell suspension. From each sample, 2.5 × 10^5^ cells (5 × 10^5^ cells/well) were pooled and added to each well of a 24-well tray and allowed to aggregate for 3–4 h on a nutator at 37°C/5% CO_2_. Three to four images of aggregated cells were taken of each replicate using a Nikon Eclipse Ti2 microscope with a 10× objective. Each experimental condition was performed with a minimum of three biological replicates with three technical repeats.

### 4.5. Mixing assay quantification

Images (10×) were acquired and exported to Fiji software where they were subjected to an equal threshold transformation, and aggregate size was assessed using the Coloc2 plugin function. The Pearson’s correlation coefficient for particle sizes from a minimum of three biological repeats (three images from three technical replicates in each) was calculated using the Coloc2 plugin. Raw data were transferred to GraphPad Prism 7 and normalized to controls: 19+13&19+13, 19+1&19+1 or 19+9&19+9, respectively. A one-way ANOVA Tukey test was performed to assess significance.

### 4.6. Immunoprecipitation

PCDH19 (pCMV-cMyc-PCDH19 or pCMV-FLAG-PCDH19) interaction with PCDH1 (pEF1a-PCDH1-cMyc-CMV-EGFP), PCDH9 (pEF1a-PCDH9-HA-CMV-EGFP) or CDH13 (pEF1a-CDH13-FLAG-CMV-EGFP) was tested by immunoprecipitation (IP) in HEK293T cells transfected with the various combinations of plasmids expressing the epitope-tagged proteins. HEK293T cells were plated at 5 × 10^5^/well in six-well culture plates. The next day, cells were transfected with the expression plasmids using Lipofectamine 3000 reagent, harvested 24 h post-transfection, lysed in cold PCDH19 lysis buffer (50 mM Tris–HCl pH 7.5, 150 mM NaCl, 0.2% Triton X-100, 2 mM EDTA, 0.01% SDS, 50 mM NaF, 0.1 mM Na_3_VO_4_ and 1× Protease inhibitor/No EDTA (Merck), sonicated (1 × 30% amplitude, 10 s on Sonic’s Vibra-Cell VCX) and centrifuged 15 000 r.p.m. at 4°C for 15 min. Lysis buffer with 0.4% Triton X-100 was used for the PCDH19–CDH13 IPs. The 10% lysate was saved as input and the remainder was subjected to IP using either EZview red anti-HA or anti-FLAG M2 agarose affinity beads (Sigma-Aldrich). IP complexes were eluted using 1× SDS protein-loading buffer (62.5 mM Tris–HCl, pH 6.8, 2% SDS, 10% glycerol and 5% *β*-mercaptoethanol [50]) at 95°C for 5 min and stored at −80°C until western blot analysis.

### 4.7. Western blotting

For western blotting, the input and IP samples were resolved by 7% homemade SDS–PAGE protein gels [50], transferred onto nitrocellulose membrane, blocked with 10% skim milk in 1× Tris-buffered saline, 0.1% Tween 20 (1× TBST), probed overnight at 4°C with appropriate primary (mouse anti-cMyc 9E10-horseradish peroxidase, mouse anti-FLAG M2, mouse anti-HA; all Sigma–Aldrich or anti-FLAG rabbit mAb; Cell signalling) and then for 2 h at room temperature with secondary antibodies (polyclonal goat anti-mouse IgG-HRP or polyclonal goat anti-rabbit IgG-HRP; both Agilent Dako) in 2% skim milk in 1× TBST. The membranes were washed with 1× TBST and the specific proteins were visualized using Clarity Western ECL substrate (Bio-Rad) on ChemiDoc XRS+ System (Bio-Rad).

## Data Availability

The raw in situ RNA seq data together with the analysis, the scripts and the raw western blotting images can be accessed using the following link: https://universityofadelaide.box.com/s/4nzwa0br5qi6xgtyaj3idda1id6by472. Electronic supplementary material is available online [51].
